# Prevalence of Metabolic Syndrome Among Individuals With Spinal Cord Injury: A Cross-Sectional Analysis

**DOI:** 10.7759/cureus.86420

**Published:** 2025-06-20

**Authors:** Seda Özcan, Belgin Erhan

**Affiliations:** 1 Physical Medicine and Rehabilitation, Uskudar State Hospital, İstanbul, TUR; 2 Physical Medicine and Rehabilitation, Faculty of Medicine, İstanbul Medeniyet Üniversitesi, İstanbul, TUR

**Keywords:** body fat percentage, cardiovascular complications, functional independence measure (fim), metabolic syndrome, national cholesterol education program adult treatment panel iii, walking index for spinal cord injury

## Abstract

Aim: This study aimed to investigate the prevalence of metabolic syndrome (MetS) among patients with spinal cord injury (SCI) and its correlation with clinical parameters.

Methods: This was a comprehensive assessment involving 100 inpatient SCI rehabilitation participants from January 2010 to December 2011. Demographic details, disease duration, and neurological evaluations following the American Spinal Injury Association (ASIA) criteria were recorded. Diagnosis of MetS adhered to the National Cholesterol Education Program Adult Treatment Panel III (NCEP-ATP III) criteria. Measurements including height, weight, waist circumference, blood pressure, and body fat percentages were taken using standardized methodologies. Biochemical analyses involved fasting blood samples for glucose, high-density lipoprotein (HDL), total cholesterol, and triglyceride levels. Functional evaluations utilized the Functional Independence Measure (FIM) for assessing daily living activities and the Walking Index for Spinal Cord Injury (WISCI) to evaluate ambulation status.

Results: A total of 42 (42%) patients had MetS. The MetS group had longer disease duration (p=0.007). Female patients showed lower waist circumference, body fat, FIM and WISCI scores, and BMI compared to male patients (p=0.01, 0.00, 0.03, 0.04, 0.04, respectively). MetS varied between tetraplegic 14% (n=3) and paraplegic patients 50% (n=39) (p=0.0059), with distinct differences in waist circumference, body fat, FIM and WISCI scores, and BMI (p=0.04, 0.00, 0.00, 0.01, 0.01, respectively). ASIA scores didn't significantly relate to MetS. While body weight differed in MetS (p < 0.0001), no associations were found with FIM and WISCI scores, height, or BMI. Patients with MetS had higher body fat (p = 0.018), but no strong links with FIM or WISCI scores (p = 0.077, 0.275, respectively).

Conclusion: SCI patients show heightened cardiovascular risks, with increasing MetS frequency linked to older age and longer injury duration.

## Introduction

Spinal cord injury (SCI) refers to damage along the spinal cord, from the base of the skull to the lower back, caused by compression, incision, or contusion. As a result, this injury disrupts the usual functions of the spinal cord beyond the injured area. Management of SCI is important both at the individual and social levels because of the accompanied psychosocial and economic problems, and its secondary complications are important causes of mortality and morbidity [1].

In the management and rehabilitation of SCI, a condition with chronic implications, prioritizing ongoing monitoring and addressing associated complications is pivotal for mitigating heightened disability and enhancing life expectancy [2]. Recent research indicates a gradual improvement in the life expectancy of individuals with SCI, eventually paralleling that of the non-injured population; therefore, managing these complications becomes even more crucial for patients [3].

Cardiovascular complications hold significant prominence within SCI patients . Of particular significance within the cardiovascular risk profile is metabolic syndrome (MetS), which significantly influences survival outcomes [4]. Individuals with SCI are prone to adopting sedentary lifestyles, which elevate the risk of developing MetS due to factors such as perivisceral obesity, glucose intolerance, insulin resistance, and reduced levels of high-density lipoprotein cholesterol (HDL-c). This sedentary behavior and pathological changes in skeletal muscle after denervation following a SCI can contribute to the emergence of various MetS risk factors, including increased abdominal fat deposition around organs, difficulties in glucose regulation, diminished sensitivity to insulin, and lower levels of the protective HDL-c [5].

Previous studies reported that determining the frequency of MetS in patients with SCI, identifying specific treatment options for patients with an increased risk, and reducing mortality, morbidity, and costs by determining cardiovascular risks can be important in prolonging survival [6]. However, only a few studies investigated the relationship between MetS development in patients with SCI and motor score or neurological classification. Although MetS has been extensively studied in the general population, data regarding its prevalence and clinical associations in individuals with SCI remain limited. Given the unique physiological and functional changes following SCI, understanding the burden of MetS in this population is crucial for early intervention and long-term health outcomes. This study was therefore conducted to address this gap and provide insight into the metabolic profile of patients with SCI undergoing rehabilitation. Within this context, this study aimed to determine the frequency of MetS in patients with SCI and its relationship with motor scores and neurological classification.

## Materials and methods

This study was conducted at Istanbul Physical Therapy and Rehabilitation Training and Research Hospital, Istanbul, Türkiye, and a total of 100 patients with SCI who had received inpatient rehabilitation at the SCI follow-up clinic between January 2010 and December 2011 were included. The study protocol was approved by the Istanbul Physical Therapy and Rehabilitation Training and Research Hospital Ethics Committee, and written informed consent was obtained from all participants.

Inclusion and exclusion criteria

Inclusion criteria were a period of at least eight weeks after SCI to regulate the blood pressure (BP) level, age >18 years, and willingness to participate in the study. Individuals under the age of 18; patients having medical conditions that may independently contribute to the development of MetS, such as Cushing's syndrome, hypothyroidism, or polycystic ovary syndrome; individuals experiencing active infections; patients presenting with comorbidities affecting ambulation (excluding those directly related to SCI, e.g., lower extremity fractures); patients diagnosed with severe psychiatric or behavioral conditions; individuals unable to comply with study procedures due to underlying issues; and those who declined to provide consent were excluded. Patients who were still in the spinal shock period were also excluded.

Data collection

Demographic data and disease duration of patients were recorded, and neurological examination was performed according to American Spinal Injury Association (ASIA) criteria [6]. The diagnosis of MetS was made according to the National Cholesterol Education Program Adult Treatment Panel III (NCEP ATP III) criteria [7]. Patients were considered to have MetS if they had three or more of the following criteria: (1) central obesity defined as waist circumference >90 cm for males and >80 cm for females, (ii) serum triglycerides >150 mg/dL or receiving treatment for hypertriglyceridemia, (iii) HDL-c <40 mg/dL for males and <50 mg/dL for females or on treatment, (iv) blood pressure >130/85 mmHg or on antihypertensive medication, (v) fasting blood glucose >100 mg/dL or receiving treatment for elevated glucose.

All anthropometric measurements were performed by a single trained evaluator using standardized protocols. Height was measured from the vertex to the heel in supine position using a stadiometer; weight was recorded in a seated position using a calibrated Sunlift 150 E scale (Sunrise Medical, Malsch, Germany). Waist circumference was measured midway between the lowest rib and the iliac crest using a flexible, non-stretchable tape during gentle expiration. Body fat percentage was obtained via whole-body dual-energy X-ray absorptiometry (DXA) (Lunar DPX-L; GE Healthcare Technologies, Inc., Chicago, Illinois, United States).

Biochemical analyses were performed by collecting blood samples after a 12-hour fasting period. All samples were centrifuged at 4°C for at least 15 minutes. The fasting blood glucose (FBG) level was measured using the glucose oxidase method. The HDL-c, total cholesterol, and triglyceride (TG) levels were calculated using the Friedewald method [8].

Functional Independence Measure (FIM) was used to evaluate the functional independence while performing activities of daily living [9]. FIM analyzes the two different aspects of disability: motor and cognitive functions. It consists of six functional areas of self-care, sphincter control, mobility, locomotion, communication, and social cognition. FIM evaluates a total of 18 activities for functional independence using a seven-point scale for each. The highest score is 126. In our study, the Turkish version of FIM was used.

The Walking Index for Spinal Cord Injury (WISCI) was used to evaluate the ambulation status of patients [10]. The WISCI is recognized for its comprehensive evaluation of walking function, encompassing diverse levels of physical support and aids essential for mobility. Its robust demonstration of face, criterion, and construct validity distinguishes it within the field​​​.

Statistical analysis

Statistical analysis was performed using Statistical Package for Social Sciences version 15.0 (Released 2007; SPSS Inc., Chicago, Illinois, United States). The Shapiro-Wilk test was used to identify group distribution. Pairwise comparisons were performed using the T-test and the Mann-Whitney U test. Pearson’s and Spearman’s correlation coefficients were used for correlation analyses. Significant difference between frequencies in groups and expected frequencies was determined using the chi-square test.

To address potential confounding factors, subgroup comparisons and correlation analyses were performed based on age, sex, and injury duration. These variables were included in the statistical analysis to explore their associations with MetS and related functional parameters. Tables were prepared using Microsoft Office Excel 2007 (Microsoft Corporation, Redmond, Washington, United States).

## Results

Of the 100 patients with SCI included in the study, 37 were female and 63 were male. The mean age of patients was 41.02±13.64 (range, 18-80) years, and the median duration of injury was 36 (range, 3-240) months. According to the ASIA impairment scale, 36 patients were classified as AIS-A, 26 as AIS-B, 17 as AIS-C, and 21 as AIS-D. A total of 21 patients were tetraplegic and 79 were paraplegic. Table 1 gives the measurements of parameters for the diagnosis of MetS. 

**Table 1:** Measurements for the diagnosis of metabolic syndrome criterion of the precipitants (N=100) HDL: high-density lipoprotein

Parameters	Range	SD
Height (cm)	155-185	7.86
Weight (kg)	46-110	13.27
BMI (kg/m^2^)	18.25-35.92	4.20
Weist circumference(cm)	72-150	15.29
Sistolic blood pressure (mmHg)	90-140	12.76
Diastolic blood pressure (mmHg)	60-95	10.36
Glucose (mg/dl)	61-349	30.52
HDL (mg/dl)	20-81	10.33

Among the participants, 42 patients had MetS. The median age of patients with MetS was 42.5 (19-80) years, and for those without MetS was 37.0 (18-73) years. The difference was not statistically significant (p = 0.087). The median injury duration in the group with MetS was 48 (4-168) months, which was statistically significantly higher than that of the group without MetS (median, 10; range, 2-240 months) (p = 0.007). MetS was diagnosed in 12 females (32%) and 30 males (48%). No significant difference was observed based on gender in the chi-square test (p = 0.202). Table 2 shows the comparison of demographic characteristics between patients with and without metabolic syndrome. 

**Table 2:** Comparison of demographic characteristics between patients with and without metabolic syndrome

Variable	With MetS (n=42)	Without MetS (n=58)	p-value
Age (years), median (range)	42.5 (19-80)	37.0 (18-73)	0.087
Injury duration (months), median (range)	48 (4-168)	10 (2-240)	0.007
Female, n (%)	12 (32%)	25 (43.1%)	
Male, n (%)	30 (48%)	33 (56.9%)	0.202

No significant difference was determined between female and male patients in respect of age, disease duration, motor and sensory scores, systolic and diastolic BP, FBG, TG, HDL, and LDL levels (Table 3; however, the waist circumference, body fat percentage, FIM, WISCI scores, and BMI values were statistically significantly lower in females (p = 0.01, 0.00, 0.03, 0.04, and 0.04, respectively). Relevant values are provided in Table 3.

**Table 3:** Comparison of clinical and metabolic parameters between female and male patients. Data are presented as mean ± standard deviation (SD). BP: blood pressure; FBG: fasting blood glucose; TG: triglycerides; HDL: high-density lipoprotein; LDL: low-density lipoprotein; SD: standard deviation.

Variable	Female (mean ± SD)	Male (mean ± SD)
Age (years)	40.78 ± 12.97	41.16 ± 14.12
Disease duration (months)	57.35 ± 62.73	45.92 ± 46.31
Motor score	54.62 ± 19.71	56.76 ± 21.5
Sensory score	131.84 ± 55.39	140.4 ± 48.35
Systolic BP (mmHg)	111.35 ± 12.51	115.71 ± 12.73
Diastolic BP (mmHg)	72.97 ± 9.39	74.76 ± 10.9
FBG (mg/dL)	89.95 ± 16.17	95.32 ± 36.39
TG (mg/dL)	133.97 ± 69.75	160.19 ± 125.17
HDL (mg/dL)	42.66 ± 11.7	39.54 ± 9.34
LDL (mg/dL)	112.54 ± 27.06	120.02 ± 31.34
Waist circumference (cm)	98.89 ± 14.86	106.73 ± 14.89

MetS was diagnosed in three (14%) patients with tetraplegia and 39 (50%) patients with paraplegia. The number of patients with paraplegia and MetS was significantly higher than those with tetraplegia (p = 0.0059). No statistically significant differences were observed in age, disease duration, FBG, TG, HDL and LDL levels between patients with tetraplegia and paraplegia, while the waist circumference, systolic and diastolic blood pressure levels, FIM and WISCI scores, and BMI were statistically significantly lower in patients with tetraplegia than those with paraplegia (p = 0.04, 0.00, 0.00, 0.01, and 0.01, respectively). Relevant values are provided in Table 4.

**Table 4:** Significant parameters according to paraplegia and tetraplegia FIM: Functional Independence Measurement; WISCI: Walking Index For Spinal Cord Injury *: p < 0.05

Parameters		Mean	SD	P value
FIM	Paraplegia	67.36	21.50	0.00*
	Tetraplegia	97.10	19.68	
WISCI	Paraplegia	2.23	5.95	0.00*
	Tetraplegia	10.73	4.34	
BMI (kg/m^2^)	Paraplegia	23.76	4.20	0.01*
	Tetraplegia	26.21	4.03	
Waist circumference (cm)	Paraplegia	94.41	12.561	0.01
	Tetraplegia	106.49	14.995	
Systolic/Diastolic BP (mmHg)	Paraplegia	107.73/67.27	11.09/ 7.673	0.04/0.00*
	Tetraplegia	115.90/ 76.03	12.68/ 10.236	

The statistical analysis on the relationship between AIS assessment scores of the patients and the presence of MetS revealed no difference between groups (p = 0.237). The group distribution is presented in Table 5.

**Table 5:** Patients with MetS according to AIS categories AIS: American Spinal Injury Association Impairment Scale; MetS: metabolic syndrome

AIS Category	MetS +, n (%)	MetS -, n (%)	Total, n
A	16 (44%)	20 (56%)	36
B	10 (38%)	16 (62%)	26
C	6 (35%)	11 (65%)	17
D	10 (48%)	11 (52%)	21

No significant relationship was observed in the FIM score, WISCI score, height, and BMI (p=0.924, 0.623, 0.805, and 0.336, respectively) between the groups with and without MetS; however, a significant difference was observed for body weight (p < 0.0001). Relationships between the FIM score, height, BMI, body weight, and MetS are provided in Table 6.

**Table 6:** Releatıonshıp of FIM and WISCI scores, height, BMI, and weight with metabolic syndrome FIM: Functıonal ındependence Measurement; WISCI: Walkıng Scale For Spınal Cord Injury; MetS: metabolic syndrome *: p < 0.05

Parameters	MetS	Mınımum		Maxımum	Mean	Standard deviation	p value
FIM	+		50	126	91.10	19.55	0.924
	-		47	126	90.17	26.17	
WISCI	+		0	20	9.57	4.21	0.623
	-		0	20	8.34	6.85	
Height (cm)	+		150	183	167.81	8.43	0.805
	-		155	185	167.41	7.50	
Weight (kg)	+		52	110	78.81	13.16	0.000*
	-		46	103	67.98	11.46	
BMI (kg/m^2^)	+		19.03	35.92	27.97	4.20	0.336
	-		18.25	33.25	24.22	3.46	

The body fat percentage was significantly higher in patients with MetS (32.8% ± 9.38%) compared to those without (27.77% ± 11.21%) (p = 0.018). A negative relationship was observed between the body fat percentage and the FIM value; however, this was not statistically significant (r 0.032, p = 0.077). A negative relationship was observed (r = −0.020) between the body fat percentage and the WISCI score, but it did not reach statistical significance (p = 0.274). The body fat percentage was negatively associated with the FIM (r = 0.032) and WISCI scores (r = −0.020), but did not reach statistical significance (p = 0.077 and 0.275, respectively).

## Discussion

This study highlights the complex interplay between MetS and clinical characteristics in individuals with SCI, particularly focusing on mobility and nutritional indicators. While gender did not significantly affect MetS prevalence, paraplegic patients exhibited a higher rate than those with tetraplegia, potentially due to differences in physical activity levels and energy expenditure. Additionally, a longer duration of injury among patients with MetS may reflect the cumulative metabolic consequences of prolonged immobility. Reduced mobility, as indicated by lower FIM and WISCI scores, may be associated with increased adiposity and altered metabolic profiles. In the current study, patients with MetS demonstrated significantly higher waist circumference and body fat percentage (p < 0.05), highlighting the link between impaired mobility, unfavorable body composition, and metabolic risk. Although BMI and height did not differ significantly between groups, the increased fat mass in the MetS group underscores the importance of evaluating nutritional status beyond standard anthropometric measures. These findings are consistent with prior research indicating that limited mobility in SCI populations contributes to increased fat accumulation and central obesity, both of which are core components of MetS. Overall, the data of the current study provide meaningful insight into the relationship between MetS, motor function, and neurologic classification in individuals with SCI.

The current study demonstrated the complex interplay between MetS and clinical parameters among SCI patients. Gender comparison showed no significant difference in MetS occurrence, but distinct variations emerged between patients with tetraplegia and paraplegia, with higher MetS prevalence in the latter. The duration of injury was notably longer in patients with MetS. Among clinical measures, waist circumference, body fat percentage, and body weight were significantly higher in patients with MetS, while no significant differences were found in height, FIM and WISCI scores, or BMI.

In this study, the frequency of MetS was determined to be 42%, which correlates with other studies in the literature. In their research exploring the correlation among fitness, inflammation, and MetS within patients with paraplegia, Manns et al. documented a MetS prevalence of 32% [11]. Similarly, in their study examining cardiovascular autonomic function among individuals with SCI, Brauman et al. identified a MetS prevalence of 52% [12]. The comparison of the results with the Turkish Adult Risk Factor (TEKHARF) study, which reported MetS prevalence in the Turkish population, showed that the frequency of MetS in SCI patients surpassed that of the healthy Turkish population across all age groups [13]. In the 30-39-year bracket, the TEKHARF study reported a 22% frequency compared to our study's 44% prevalence. Correspondingly, in the 40-49, 50-59, and 60-69-year age groups, the current study indicated higher frequencies compared to the TEKHARF study: 40% vs. 46%, 46% vs. 52%, and 47% vs. 62%, respectively. These findings underscore the heightened prevalence of MetS within individuals with SCI compared to the general population and highlight the significance of monitoring MetS in the context of complications and the rehabilitation trajectory for these patients.

The development of MetS was found to be associated with advanced age and increased disease duration in this study. Also, the frequency of MetS was higher in our male patients compared to that in the Turkish adult population within the same age group [13] on average (48% vs. 40%, respectively) and lower in our female patients (32% vs. 39%, respectively). Similar to our findings, Grundy et al. reported that a 10-year increase in age leads to a 1.5 times higher chance of having MetS in patients with SCI [14]. 

Understanding gender-based variations in body fat distribution and their connection to heart-related metabolic issues and pro-inflammatory substances in SCI remains limited [15]. For example, Jones et al. [16] reported that men with SCI have a greater occurrence of MetS compared to able-bodied men, while Liang et al. [17] reported that men with SCI do not seem to show a higher prevalence of MetS compared to their able-bodied counterparts. More comprehensive studies involving larger patient populations are needed in this field.

The study findings showed that women had lower BMI values compared to men, which may have contributed to the lower prevalence of MetS observed in female patients. Groah et al. conducted a study that examined individuals with SCI who were of similar ages and genders [18]. Through their investigation, it was found that patients with SCIs typically consumed fewer calories than the recommended daily intake. Interestingly, female patients with SCIs showed a preference for lower-calorie foods, while their male counterparts tended to gravitate towards higher fat consumption. In addition, other factors like lower FIM and WISCI scores may play a role in women experiencing compromised nutritional status compared to men. These scores assess self-feeding abilities, ambulation, and functional independence.

It was found that patients with tetraplegia had a lower daily calorie intake compared to individuals with paraplegia. This difference may be partially explained by reduced functional independence [19,20]. The difference in calorie intake may be a factor in the lower BMI, smaller waist circumference, and lower occurrence of MetS among patients with tetraplegia compared to those with paraplegia. This may suggest an association with nutritional insufficiency. In addition, the blood pressure levels in SCI patients with tetraplegia are lower, which is in line with what we would expect due to autonomic dysfunction. This raises the question of whether standard diagnostic criteria for MetS are fully applicable to this specific population.

Investigating the connection between MetS development in patients with SCI and neurological factors, Liang et al. discovered that the frequency of MetS remained unchanged regardless of the neurological level and completeness status [17]. It appears that there are other factors to consider besides the loss of motor function, which should be thoroughly examined. While reduced mobility is generally considered a contributing factor to metabolic risk, our findings did not show a direct association between FIM/WISCI scores and MetS occurrence. This may reflect limitations of current functional scales in capturing total physical activity or metabolic demand [20,21]. It is crucial to take into account factors beyond just ability, such as daily exercise capacity and functional independence levels, when evaluating the relationship between MetS and physical performance. Prior studies have highlighted the importance of developing better measurement tools to assess the physical activity levels of individuals with SCI, particularly when it comes to tracking the duration of active exercise and higher-intensity activities [17]. The relationship between physical activity and MetS development is still not fully understood, and the findings from Lee et al. [22] have added to the complexity. They found no connection between functional independence and inflammatory cytokine levels. This emphasizes the complex factors involved in MetS, going beyond just physical activity and obesity, which have traditionally been seen as the main causes.

Our study analyzed obesity indicators, such as BMI and body fat percentage, in patients with SCI. The results revealed a mean BMI of 25.79 ± 4.20, with 14% of the patients classified as obese (BMI > 30%). Nevertheless, there was no notable disparity in BMI observed between the patients with MetS and those without it. Other studies have also found similar results, indicating that there are additional factors besides obesity that play a role in MetS among individuals with SCI [17]. The current study also unveiled a significant discrepancy in body fat percentage between patients with and without MetS. This finding challenges the reliability of BMI as an obesity indicator in SCI patients, attributing this inaccuracy to altered fat distribution [23]. The findings emphasize that it could be of benefit to employ more precise measurement techniques, such as DXA, for body fat distribution.

The findings of this study showed an association between longer duration post SCI and increased prevalence of MetS. Notably, sex and injury level seemed to influence the lower occurrence of certain conditions in female patients and those with tetraplegia, possibly due to compromised nutritional status stemming from diminished functional levels in these groups. Interestingly, functional independence and ambulation levels did not significantly impact MetS development in the study. This intriguing observation suggests that genetic susceptibility and yet unidentified inflammatory mechanisms may also play a role. This suggests a need for further investigation to unravel these complexities and their roles in MetS among SCI patients.

There are some limitations to consider in this study. Key metabolic and inflammatory markers such as insulin resistance, C-reactive protein, and interleukin-6 were not measured. Additionally, detailed assessments of nutritional status and daily physical activity levels, important contributors to metabolic health, were not included. Furthermore, several potential confounding variables, such as medication use (e.g., corticosteroids), dietary intake, hormonal status, and inflammation, were not included in the analysis. This may affect the interpretation of causality and the generalizability of our findings. These omissions may limit the interpretation of underlying mechanisms related to MetS in individuals with SCI. Future studies with larger and more diverse populations should aim to include comprehensive biological, nutritional, and lifestyle data. Furthermore, exploring the clinical implications of these findings may contribute to the development of more targeted preventive and therapeutic strategies in this population.

## Conclusions

This study demonstrates a notably high prevalence of MetS among individuals with SCI undergoing inpatient rehabilitation. By integrating metabolic parameters with functional measures such as FIM and WISCI, we provide a comprehensive evaluation of patients’ cardiometabolic and functional profiles. These findings underscore the necessity of implementing systematic metabolic screening protocols in the clinical follow-up of SCI patients. Furthermore, early intervention strategies targeting modifiable risk factors may potentially improve long-term cardiovascular outcomes and enhance functional recovery. Future prospective multicenter studies are warranted to establish causal relationships and to develop evidence-based guidelines tailored to this vulnerable population.
